# The clinical impact of MRI screening for BRCA mutation carriers: the first report in Japan

**DOI:** 10.1007/s12282-019-00955-6

**Published:** 2019-02-28

**Authors:** Wakana Murakami, Mitsuhiro Tozaki, Seigo Nakamura, Yoshimi Ide, Mayuko Inuzuka, Yuko Hirota, Kouzou Murakami, Noritsugu Takahama, Yoshimitsu Ohgiya, Takehiko Gokan

**Affiliations:** 1 Department of Radiology, Division of Radiology, Showa University Graduate School of Medicine, 1-5-8 Hatanodai, Shinagawa-ku, Tokyo, Japan; 2 Department of Radiology, National Defense Medical College, 3-2 Namiki, Tokorozawa, Saitama Japan; 3 Department of Radiology, Sagara Hospital Affiliated Breast Center, 3-28 Tenokuchi-cho, Kagoshima, Kagoshima Japan; 4 Department of Radiology, Division of Radiology, Showa University School of Medicine, 1-5-8 Hatanodai, Shinagawa-ku, Tokyo, Japan; 5 Department of Surgery, Division of Breast Surgical Oncology, Showa University School of Medicine, 1-5-8 Hatanodai, Shinagawa-ku, Tokyo, Japan; 6 Breast Center, Showa University, 1-5-8 Hatanodai, Shinagawa-ku, Tokyo, Japan; 7 Department of Pathology, Showa University School of Medicine, 1-5-8 Hatanodai, Shinagawa-ku, Tokyo, Japan; 8 Department of Radiology, Division of Radiation Oncology, Showa University School of Medicine, 1-5-8 Hatanodai, Shinagawa-ku, Tokyo, Japan

**Keywords:** BRCA, High-risk patients, Familial breast cancer, MRI surveillance, Mammography

## Abstract

**Background:**

There is no consensus on the appropriate surveillance for high-risk women with breast cancer in Japan. We investigated their imaging features and pathological characteristics to build a proper surveillance system for asymptomatic high-risk individuals in the future.

**Methods:**

We retrospectively reviewed 93 female (median age 43 years) BRCA1 and BRCA2 mutation carriers from our institutional clinical database from 2011 to 2017. The study population was composed of 112 breast cancers. Mammography and MRI were reviewed by examiners blinded to patients’ clinical history. Final surgical or biopsy histopathology served as the reference standard in all the patients.

**Results:**

Fifty-nine breast cancers met selection criteria; of these, 30 were BRCA1-associated tumors, and 29 were BRCA2-associated tumors. Invasive ductal carcinoma was the most prevalent type in both BRCA1 and BRCA2. There were statistically significant differences in phenotype, nuclear grade, and Ki-67 labeling index between BRCA1 and BRCA2 mutation carriers. Additionally, imaging findings on mammography and MRI were statistically different. Tumors in BRCA2 carriers demonstrated mammographic calcifications more frequently, while those in BRCA1 carriers demonstrated a mass or architectural distortion (*P* < 0.001). Enhancement pattern on MRI also significantly differed between the two subgroups (*P* = 0.006). The size of MRI-detected lesions was statistically smaller than the size of those detected by other modalities (*P* = 0.004).

**Conclusions:**

The imaging and histological characteristics of BRCA1/2 mutation carriers were consistent with other countries’ studies. MRI-detected lesions were significantly smaller than lesions detected by non-MRI modality. All lesions in BRCA1 mutation carriers could be detected by MRI.

## Introduction

Currently, in Japan, women of average risk for breast cancer are advised to undergo mammography every 2 years beginning at age 40 years. However, no suitable surveillance system for women at high risk of developing breast cancer has yet been established.

Sugano et al. [1] reported that the prevalence of BRCA1/2 mutations in the Japanese population was higher than in non-Ashkenazi individuals under the same conditions of personal and family history of breast and/or ovarian cancer (odds ratio 1.87). A number of countries other than Japan have previously conducted studies that demonstrated differences in histopathology and imaging characteristics between breast cancers associated with BRCA1 and BRCA2 mutations [2, 3]. Hence, it is critical to work on establishing surveillance systems for high-risk women that are reflective of the epidemiology in Japan.

Although the Guidebook for Diagnosis and Treatment of Hereditary Breast and Ovarian Cancer Syndrome 2017, an initiative spearheaded by Japan’s Ministry of Health, Labor, and Welfare, recommends annual contrast-enhanced MRI examination for women aged ≥ 25 years who have a BRCA mutation, currently, this screening study is not covered by the national health insurance program. In April 2018, the national health insurance coverage of MRI-guided biopsy was instituted, allowing more ready access to MRI-guided biopsy for high-risk patients, which is consistent with guidelines of other countries, such as the American Cancer Society Guideline for Breast Screening with MRI as an Adjunct to Mammography (2007) (ACS 2007) [4] and the National Comprehensive Cancer Network (NCCN) Guidelines Version 1. 2018 for Genetic/Familial High-Risk Assessment (http://www.nccn.org).

Consequently, the present study aimed to investigate imaging features of breast cancer developing in Japanese BRCA1/2 mutation carriers to build a proper surveillance system for asymptomatic high-risk individuals in the future.

## Materials and methods

### Study population

This single-institution retrospective study was approved by the institutional review board of Showa University.

A total of 112 consecutive breast cancer lesions in 93 patients (all female; median age 43 years) from 90 families were extracted from the database of patients who had tested positive for BRCA1 or BRCA2 at our hospital between January 2011 and March 2017. Of the cases identified, 53 breast cancer lesions had incomplete image-based assessment and were, therefore, excluded; mammography was not performed for 49 lesions, and MRI was not performed for 40 lesions, and neither imaging modality was used for 36 lesions. This study, therefore, included 59 breast cancer lesions (from 52 distinct patients) which had been imaged by both mammography and MRI. Simultaneous and metachronous bilateral breast cancers were identified in 7 of the 52 patients (13.5%).

Genetic testing was performed only after imaging screening for 51 patients. Essentially, these patients did not undergo imaging for high-risk purposes. Only one patient underwent imaging as hereditary breast and ovarian cancer (HBOC) surveillance screening.

### Patient and tumor features

The following patient and tumor features were recorded: age at the time of cancer diagnosis, presenting scenario prompting tumor discovery, BRCA mutation type, histology, phenotype, nuclear grade, Ki-67 labeling index, and lymph node status.

Final surgical or biopsy histopathology served as the reference standard in all the patients.

### Imaging acquisition and analysis

Breast MR imaging in our facility was performed with the patient in the prone position on 1.5-T system (SIGNA HDxt; GE Healthcare, Waukesha, Wis) using a dedicated eight-channel surface breast coil. Each patient received 0.1 mL gadobutrol (Gadovist; Bayer Schering Pharma, Berlin, Germany) per kilogram of body weight as a contrast agent, injected at a rate of 1 mL/s. Axial T1-weighted images (repetition time ms/echo time ms 6.1/2.9, matrix 384 × 256, section thickness 1.6 mm, flip angle 10°, FOV 350 mm), axial T2-weighted images (repetition time ms/echo time ms 3800/98, matrix 448 × 256, section thickness 2 mm, flip angle 90°, FOV 350 mm), axial fat-suppressed T2-weighted images (repetition time ms/echo time ms 4400/98, matrix 448 × 256, section thickness 2 mm, flip angle 90°, FOV 350 mm), and diffusion-weighted images (*b* values = 0, 1000 s/mm^2^; repetition time ms/echo time ms 10,000/86, matrix 128 × 128, section thickness 5 mm, flip angle 90°, FOV 350 mm) were acquired. Dynamic contrast–enhanced MR imaging included one pre-contrast and three post-contrast acquisitions with bilateral axial acquisition and fat-suppressed T1-weighted imaging.

The mammograms and MR images of the subjects who had been entered in our facility’s database were reviewed retrospectively by two breast radiologists (with 2 and 25 years of experience), who were blinded to the patient’s clinical information and other modality information. This image database also included images from some other medical centers. The mammography images were divided into four groups based on the BI-RADS breast density scale of the American College of Radiology (ACR) [5] (entirely fatty, fibroglandular dense, heterogeneously dense, and extremely dense), and findings were recorded for the presence of microcalcification and mass and/or architectural distortion. On the MR images, we measured tumor size (diameter or extent) at the time of diagnosis. The enhancement pattern was studied to determine if it was a mass or non-mass enhancement. Mass lesions were also studied for a time intensity curve pattern and peritumoral edema or the absence thereof. The locations of the lesions were also divided into anterior, middle, posterior, and whole. Final assessment categories were classified as indicated in the ACR BI-RADS atlas.

We also compared the tumor size on MRI at the time of diagnosis according to three patient presentation groups. Cases were divided into self-detected; detected on mammography or ultrasonography (US) (by routine-screening exams on mammography, US, or both and by post-operative contralateral follow-up); and detected on MRI (by post-operative contralateral follow-up and by HBOC surveillance exam). The routine-screening exams were conducted as part of the public service cancer screening program in Japan.

### Statistical analysis

All statistical analyses were performed using software R version 3.2.3 [6]. The Wilcoxon signed-rank test was used to compare the age and tumor size between BRCA1 and BRCA2 mutation carriers. Fisher’s exact test was used to compare tumor histological features and imaging findings on mammography and MRI across mutational subgroups. Moreover, Kruskal–Wallis test was used to compare the size depending on patient presentation. A *P* value of < 0.05 was regarded as statistically significant between the groups.

## Results

### Patients and tumor features

Fifty-nine breast cancers were documented. Seven patients with bilateral breast cancers include four patients with simultaneous bilateral cancers and three patients with contralateral metachronous breast cancers. The simultaneous group included two patients who underwent contralateral risk-reducing mastectomy (CRRM) and were incidentally found to have contralateral ductal carcinoma in situ (DCIS). The median interval period in the metachronous group was 5 years between the first breast cancer and the contralateral breast cancer.

Details of patients and tumor features are shown in Table 1. The tumors which had been imaged by both mammography and MR were divided into 30 breast cancers in BRCA1 mutation carriers and 29 breast cancers in BRCA2 mutation carriers. The median patient age was 40 years (range 25–71 years) in BRCA1 mutation carriers and 43 years (range 23–73 years) in BRCA2 carriers (*P* = 0.660). The subjects had lower ages at onset than the age at which breast cancer tends to occur in the general population. Invasive ductal carcinoma was the most prevalent histology in both groups; however, the distribution of tumor histology differed significantly between BRCA1 and BRCA2 mutation carriers (*P* = 0.002). Tumors in BRCA1 and BRCA2 mutation carriers also demonstrated statistically significant differences in phenotype, nuclear grade and Ki-67 labeling index (*P* < 0.001, *P* = 0.020, *P* = 0.001, respectively). Tumors in BRCA1 mutation carriers were associated with significantly higher histological and nuclear grade, or Ki-67 status. While DCIS was not detected in BRCA1 carriers, the histology of specimens from BRCA2 mutation carriers frequently contained DCIS. No significant difference was observed for lymph node status.

**Table 1:** Patient and tumor features

Variables		No. of BRCA1 carriers (*n* = 30)	No. of BRCA2 carriers (*n* = 29)	*P* value
Age (median, range)		40 (25–71)	43 (23–73)	0.660
Histology				0.002*
IDC		29 (96.7)	18 (62.1)	
ILC		0	1 (3.4)	
Others^a^		1 (3.3)	3 (10.3)	
DCIS		0	7^b^ (24.1)	
Phenotype				< 0.001*
Luminal		3 (10.0)	20 (69.0)	
Luminal HER2		0	0	
HER2 enriched		1 (3.3)	0	
Triple negative		26 (86.7)	7 (24.1)	
N/A		0	2 (6.9)	
Nuclear grade				0.020*
1, 2		4 (13.3)	12 (41.4)	
3		14 (46.7)	7 (24.14)	
N/A		12 (40.0)	10 (34.5)	
Ki-67 labeling index				0.001*
≥ 20%		24 (80.0)	14 (48.3)	
< 20		2 (6.7)	14 (48.3)	
N/A		4 (13.3)	1 (3.4)	
Lymph node status				1.000
Positive		18 (60.0)	17 (58.6)	
Negative		7 (23.3)	8 (27.6)	
N/A		5 (16.7)	4 (13.8)	

*IDC* invasive ductal carcinoma, *ILC* invasive lobular carcinoma, *DCIS* ductal carcinoma in situ, *HER2* human epidermal growth factor receptor 2, *N/A* not available

*Statistically significant

^a^Includes metaplastic carcinoma and mucinous carcinoma

^b^Includes two cases diagnosed by CRRM (contralateral risk-reducing mastectomy)

### Imaging features

MRI evaluation was performed for 72 breast cancer lesions. The size of lesions detected on MRI was statistically smaller than the size of those detected on other modalities (*P* = 0.004). In this analysis, 12 cases were excluded; two cases were examined for pre-operative contralateral evaluation, one case was incidentally detected on chest CT obtained for another purpose, two patients underwent MRI after vacuum-assisted breast biopsies, two breast cancers were revealed by CRRM, and five patients’ presentations were unknown in our database. Therefore, a total of 60 lesions were analyzed (Table 2). Of the 60 lesions, 40 (66.7%) were patient detected. They exhibited a palpable mass, pain, or nipple discharge without any prior routine-screening exams or interval screening exams. Tumor size of self-detected lesions significantly differed from that of mammography and US-detected lesions (*P* < 0.001). In addition to this, the size of the lesion detected by MRI based on the three groups (self-detected, detected on mammography or US, and detected on MRI) differed significantly (*P* = 0.002). Furthermore, when analysis was limited to the mass lesions (with non-mass enhancement excluded), the difference met a stricter significance threshold (*P* = 0.001).

**Table 2:** Tumor size according to patient presentation

Patient presentation	Tumor size on MRI (mm)			*P* value	Tumor size on MRI (mm)			*P* value
	[Mass + non-mass]				[Mass only]			
		Median	Range	0.002*		Median	Range	0.001*
Self-detected^a^	(*n* = 40)	28	(11–82)		(*n* = 39)	28	(16–82)	
Detected on MMG or US								
Detected by routine-screening exams^b^	(*n* = 15)	21	(9–110)		(*n* = 12)	23	(9–110)	
Detected by post-operative contralateral follow-up	(*n* = 2)	17.5	(13–22)		(*n* = 2)	17.5	(13–22)	
Detected on MRI								
Detected by post-operative contralateral follow-up	(*n* = 2)	4	(3–5)		(*n* = 2)	4	(3–5)	
Detected by HBOC surveillance exam	(*n* = 1)	7			(*n* = 1)	7		
Detected on other than MRI for some purposes^c^	(*n* = 3)	19	(17–57)		(*n* = 2)	18	(17–19)	
Undetectable on MRI^d^	(*n* = 4)	0						
Unknown	(*n* = 5)							

*MMG* mammography, *US* ultrasonography, *HBOC* hereditary breast and ovarian cancer

*Statistically significant

^a^Patients who presented with clinical symptoms (i.e., mass, pain, or nipple discharge); these patients had no prior routine-screening exams, or symptoms occurred between interval screening exams

^b^Publicly available routine cancer screening in Japan

^c^These included two cases for pre-operative contralateral evaluation and one case detected on chest CT incidentally

^d^Two patients underwent MRI after US-guided vacuum-assisted breast biopsies, and two breast cancers were diagnosed by CRRM (contralateral risk-reducing mastectomy)

**Fig. 1:**
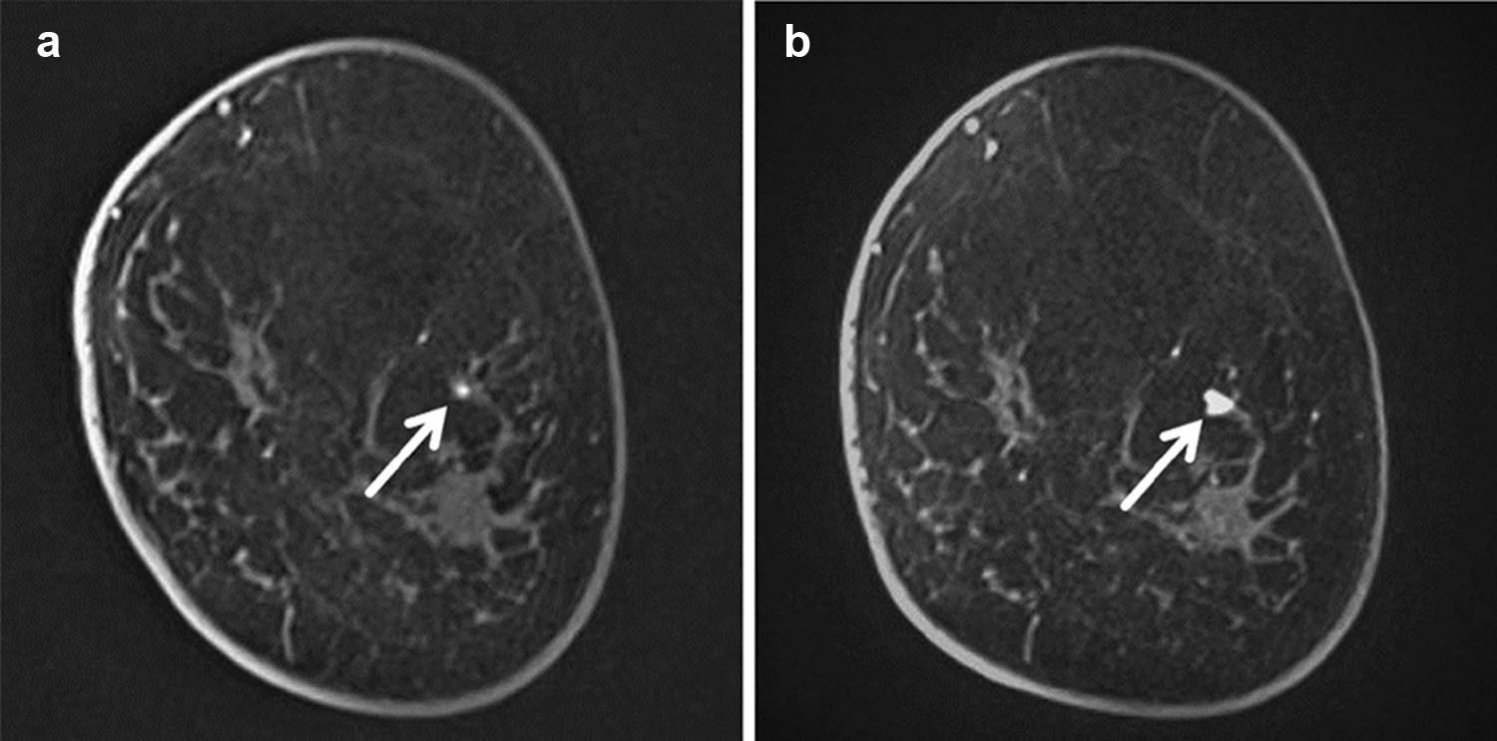
**a, b** Coronal first dynamic contrast-enhanced MR image of left breast in 35-year-old female BRCA1 mutation carrier. These MR images were performed for post-operative contralateral follow-up as she underwent surgery for the right breast at the age of 32. A new focus lesion was detected in 3 mm in diameter on MR surveillance (arrow of **a**). After observation for 6 months, it increased to 7 mm in diameter (arrow of **b**). As this lesion could not be detected on second-look ultrasound, MRI-guided biopsy was performed. It was diagnosed as triple-negative ductal carcinoma in situ (DCIS)

**Fig. 2:**
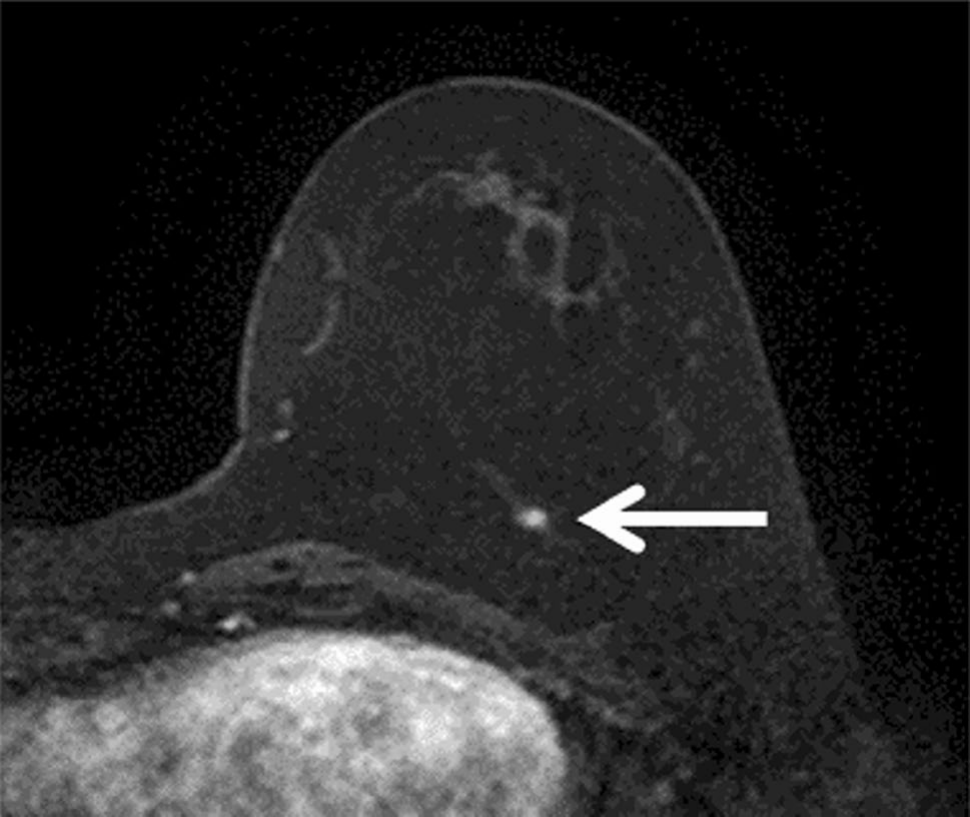
Transverse first dynamic contrast-enhanced MR image in an asymptomatic 52-year-old female BRCA1 mutation carrier for post-operative follow-up evaluation, which revealed a 5 mm mass lesion (arrow) in the left breast. Through MRI-guided biopsy, this case was diagnosed as invasive ductal cancer, triple-negative

We noted three tumors in with BRCA1 mutation carriers detected by MRI only. Two of these lesions were 3 mm (Fig. 1a) and 5 mm (Fig. 2) in diameter on post-operative contralateral follow-up MRI. The 3 mm lesion was observed for 6 months and seen to grow to 7 mm, prompting an MRI-guided biopsy at that time, as the lesion was not sonographically visualized (Fig. 1b). The third MRI-detected BRCA1-associated lesion was found during the first round of HBOC surveillance. This HBOC surveillance exam was performed because of the patient’s strong family history.

Detection rates on mammography and MRI at the time of the diagnosis according to breast density in mutation carrier subgroups are illustrated in Table 3. The reference standard was the result of the pathology examination. Two cases of BRCA2 mutation carriers were excluded because MRI evaluations were performed after vacuum-assisted breast biopsies. All BRCA1-associated lesions were detectable by MRI, while two cases of BRCA2-associated lesions could not be detected by MRI. These two BRCA2 lesions were also undetectable on mammography. They were incidentally found by CRRM and diagnosed as DCIS. Though we tried to evaluate those lesions again in this study, both could not be detected using mammography and MRI. The detection rate differed between mammography and MRI, although this difference did not reach statistical significance (84.7% [50/59] vs 96.5% [55/57], respectively; *P* = 0.053). We also found that patients with negative mammography tended to have dense breasts.

**Table 3:** Detection rates on mammography and MRI

Density on MMG	BRCA1 (*n* = 30)		BRCA2 (*n* = 29)	
	Detection rate of MMG	Detection rate of MRI	Detection rate of MMG	Detection rate of MRI
Entirely fatty	None	None	None	None
Fibroglandular density	80.0 (4/5)	100.0 (5/5)	100.0 (2/2)	100.0 (2/2)
Heterogeneously dense	84.6 (11/13)	100.0 (13/13)	92.9 (13/14)	100.0 (12/12)^a^
Extremely dense	75.0 (9/12)	100.0 (12/12)	84.6 (11/13)	84.6 (11/13)^b^

^a^Two BRCA2 tumors were excluded because MRI evaluations were performed after vacuum-assisted breast biopsies

^b^Two lesions which were undetectable by MRI were diagnosed by contralateral risk-reducing mastectomy

For imaging characteristics of mammography and MRI, significant differences were observed between the two subgroups (Table 4). Twenty-four of 30 (80%) BRCA1 lesions, which were all depicted on mammography, manifested as a mass and/or architectural distortion on mammography. On the other hand, BRCA2 lesions tended to exhibit microcalcification; 16 of 26 (61.5%) BRCA2 lesions appeared as microcalcification alone or in combination with other findings. We detected a significant difference in the distribution ratio of mass-to-non-mass lesions on MRI between them (*P* = 0.006), though tumor size did not differ significantly (*P* = 0.994). All BRCA1 tumors manifested as a mass on MRI. However, findings of the time intensity curve on dynamic contrast-enhanced MRI and peritumoral edema on T2-weighted images did not significantly differ according to the BRCA mutation status. Our study demonstrated that BRCA1 tumors tended to occur at posterior areas, though no significant difference was observed in regional distribution on MRI between BRCA subgroups (*P* = 0.180).

**Table 4:** Imaging findings on mammography and MRI

Imaging findings	No. of BRCA1 carriers (*n* = 30)		No. of BRCA2 carriers (*n* = 29)	*P* value
Mammography				< 0.001*
Microcalcification	0		13 (50.0)	
Mass and/or architectural distortion	23 (95.8)		10 (38.5)	
Both	1 (4.2)		3 (11.5)	
Total no. of detected lesions	24		26	
MRI				
Size (median, range)	25.5 (5–82)		26 (0–110)	0.994
Mass	30 (100.0)		19 (76.0)	0.006*
Non-mass enhancement	0		6 (24.0)	
Total no. of detected lesions	30		25^a^	
Time intensity curve of mass lesions				0.396
Fast-persistent	10 (33.3)		4 (16.0)	
Fast-plateau	2 (6.7)		2 (8.0)	
Fast-washout	14 (46.7)		14 (56.0)	
N/A	4 (13.3)		5 (20.0)	
Peritumoral edema	16 (53.3)		13 (68.4)	1.000
Lesion location				0.180
Anterior	2 (6.7)		1 (4.0)	
Middle	2 (6.7)		7 (28.0)	
Posterior	24 (80.0)		15 (60.0)	
Whole	2 (6.7)		2 (8.0)	

^a^Four lesions could not be detected on MRI at the time of diagnosis because MRI evaluation for two of them performed after US-guided vacuum-assisted breast biopsies and the other two lesions were incidentally diagnosed by contralateral risk-reducing mastectomy (CRRM)

*Statistically significant

## Discussion

The most significant finding of the current study is the difference in size between the lesions detected by MRI and the lesions detected by other modalities (5 mm vs 26 mm; *P* = 0.004). Of MRI-detected lesions, two cases were diagnosed by MRI-guided biopsy; one patient underwent both MRI screening exam and MRI-guided biopsy at her own expense because it was not covered by national health insurance yet at that time, as only since April 2018 has MRI-guided biopsy been covered by the national health insurance; the other lesion was the first case reported as having undergone MRI-guided biopsy for high-risk patients through coverage of the national health insurance. On the other hand, the third case was visible on second-look US and diagnosed by US-guided biopsy. A similar such case of HBOC surveillance was reported by Tozaki et al. [7]. These two cases will be part of a larger surveillance study, which is supported by the Health Labor Science Research Grant and is still ongoing. The MRI-detected lesions were all associated with BRCA1 mutation. These cases may not be unusual in countries outside of Japan where “quality-assured MRI screening” [8] is standard of care for high-risk patients. Guidelines for this screening modality are outlined in the ACS 2007 [4] and NCCN Guidelines Version 1. 2018 for Genetic/Familial High-Risk Assessment (http://www.nccn.org). As part of high-quality breast MRI screening, ability to perform biopsy under MRI guidance is a required condition.

In Japan, however, the studies of MRI-guided biopsy were reported only by Tozaki et al. [9, 10] and this study is the first report about cases of MRI-guided biopsy performed for high-risk patients. Due to the lack of coverage by national health insurance, MRI-guided biopsy was not widely accessible before April 2018. Therefore, most of the facilities generally performed US-guided biopsy for MRI-detected lesions. As found by Sakamoto et al. [11], the false-negative rate of US-guided biopsy for MRI-detected lesions (26%; 8/31) was significantly higher than that for US-detected lesions (3.5%; 5/144) (*P* = 0.0002). They concluded that MRI-guided biopsy was required for MRI-detected lesions.

According to the most recent Japanese Breast Cancer Society Clinical Practice Guidelines for Breast Cancer released in 2018, CRRM is strongly recommended for patients who have developed breast cancer associated with HBOC. This guideline was not available before the initiation of national insurance coverage of MRI-guided biopsy. It is necessary to review the utility of MRI-guided biopsy from the viewpoint of cost-effectiveness in the current environment when it is covered by the national health insurance. In addition to the guidelines regarding CRRM, we further recommend MRI screening and MRI-guided biopsy, given that they improve early detection as evidenced by the fact that the three MRI-detected lesions in our series were very small, at an average size of 5 mm.

Our study included two prophylactic mastectomies with BRCA2 cancers, which were diagnosed as DCIS (4 mm and 8 mm in extent, respectively). They were both undetected by any modalities during pre-operative evaluation. Findings of previous studies showed that most of the lesions diagnosed by CRRM were DCIS [12]. It is important for high-risk women to obtain diagnosis by excision of tumors at an early stage such as DCIS. On the other hand, one of the three MRI-detected lesions was diagnosed as DCIS with the size of 3 mm in our study. Though this result does not prove that MRI screening surveillance is superior to CRRM, we may consider providing this option to patients as well as CRRM. Previous studies indeed suggested MRI surveillance might be more suitable for the detection of contralateral breast cancer compared with other modalities [13, 14].

These features have been discussed in the past, as breast cancer with BRCA1 mutation carriers has the tendency to develop rapidly growing triple-negative cancer with poor prognosis [2, 15]. Rijnsburger et al. [16] also showed that results in the BRCA1 group were worse compared to the BRCA2 for mammography sensitivity, tumor size at diagnosis, proportion of DCIS and interval cancers, and age at diagnosis. As the result of Japanese data by Nakamura et al. [17], the proportion of triple-negative cancer in BRCA-positive tumors was larger than that of BRCA2-positive tumors (BRCA1, 62.2% [23/37]; BRCA2, 14.3% [5/35]). The result of Vreeman et al. [18] demonstrated that MRI surveillance is insufficient for BRCA1 mutation carriers because the sensitivity of a combined annual MR examination with mammography breast cancer screening program in BRCA1 mutation carriers was the lowest (81.3%) compared with other groups (BRCA2, 92.0%; family history, 95.2%; personal history, 91.1%). The previous study by Kuchenbaecker et al. [19] on contralateral breast cancer showed a 20-year cumulative risk after breast cancer diagnosis in 40% of BRCA1 carriers and 26% of BRCA2 carriers (hazard ratio for comparing BRCA2 vs BRCA1, 0.62; 95% CI 0.47–0.82; *P* = 0.001). These results suggest the need for a tailored management plan based on the risk stratification of the high-risk groups in Japan. CRRM might be recommended for BRCA1 cancers, while BRCA2 cancers may avoid CRRM through the use of thorough MRI surveillance and improvements in the indication and the technical skills required in MRI-guided biopsy. The data gleaned from the three cases of MRI-detected lesions in BRCA1 mutation carriers discussed in our study would be valuable in developing such a surveillance plan in Japan.

BRCA1 tumors did not present as microcalcifications on mammography, but rather as masses on MRI. In addition, no DCIS was observed in BRCA1-positive cancers. As reported by Kuhl et al. [20], BRCA1 tumors tend to grow rapidly, and as a result, having imaging characteristics of a benign tumor, such as a fibroadenoma or cyst. It is, therefore, crucial for radiologists to be vigilant in the imaging diagnosis, as well as the management decisions regarding proper follow-up interval for surveillance. For BRCA2 mutation carriers, however, six lesions showed non-mass enhancement, and all these involved microcalcification with luminal subtype. Four of these cases were diagnosed as DCIS. Thus, the fact that while BRCA1- positive tumors have potentially aggressive characteristics to develop triple-negative cancers, BRCA2-positive tumors are more likely to present as luminal type or DCIS, which is reflected in the imaging features of these lesions for our study population, a finding that is consistent with the previous studies [2, 3].

Based on our results, the sensitivity of mammography was 84.7% (50/59). Such high sensitivity is also consistent with the results of previous studies [2, 3]. However, other studies reported lower mammography sensitivity [21, 22]. Ha et al. [2] reported that high detectability on mammography might be partly due to the stage of patients with breast cancer registered in the study. Relatively large size at the time of diagnosis on MRI was indeed common in the current study, as shown in Table 4. Another possible explanation for the observed high sensitivity of mammography may relate to diagnostic suspicion bias in these retrospective studies.

Mammography as surveillance for high-risk patients requires discussion about the age at which mammography surveillance is recommended and potential benefits. As shown in NCCN guidelines, the combination of MRI and mammography is recommended for high-risk patients older than 30 years, which is based on a study using a computer simulation modeling [23]. Mammography indeed makes a significant contribution through its ability to detect microcalcifications. Phi et al. [24] reported that mammography’s specific ability to detect microcalcifications is especially valuable in BRCA2 mutation carriers because the proportion of calcified DCIS in women who are BRCA2 mutation carriers was larger than that of women who are BRCA1 mutation carriers.

Two additional issues about the significance of mammography warrant further discussion. The first is the issue about breast density, and the second is about pathological grade. The strong tendency of high-risk groups to have dense breast tissue is known [25]. The rate of Japanese women with dense breasts is especially higher than Western people [26–28]. In addition, it has been suggested that breast density is associated with the risk of breast cancer [29, 30]. Indeed, the rate of dense breasts in our study was relatively higher than the rate found in previous Western studies about imaging features of high-risk women [3, 20]. Sawada et al. [26] also reported that the false-negative rate for Japanese women with dense breasts was high. For our current study, in all the cases where mammography was unable to detect the lesions, the breast density was described as either heterogeneous or extreme density. Furthermore, our results did not include any breast cancer cases that were MR negative and mammography positive. Previous studies also demonstrated that the sensitivity of MRI is superior to that of mammography for follow-up in high-risk groups [8, 16, 21, 31–34]. The result of the study by Kuhl et al. [35] showed that 83% of the cases of only mammography-positive DCIS were non-high grade although 60% of DCIS diagnosed by MRI alone were high grade. They also suggested that some of the cases of DCIS diagnosed by mammography were biologically benign and would never threaten a woman’s life. As the result of other studies [34, 36, 37], BRCA mutation carriers younger than 40 years may not benefit from mammography in addition to MRI, because they rarely develop MRI-occult breast cancers. We must also not forget the critical issue of the susceptibility to the mutagenic effects of radiation [38]. According to the EVA trial [8], which was a prospective multicenter observational cohort study looking at 687 women at elevated familial risk, women undergoing quality-assured MRI annually would not require mammography, ultrasound, or even clinical breast exam. MRI alone was sufficient for cancer detection, especially in the preinvasive stage. In the future, larger population-based studies evaluating the added value of mammography will be needed for the development of a surveillance system and risk models in Japan.

The current study demonstrated that lesions associated with BRCA mutation carriers tended to develop in the posterior portion of the breast (Table 4). This result is also consistent with the study by Schrading et al. [20] and Ha et al. [2]. Triple-negative breast cancers have a tendency toward a posterior portion [39]. We believe the results of these reports are related to the tendency of BRCA1 mutation carriers to develop triple-negative cancers as shown earlier. However, the mechanisms for the skewed localization was not completely provided in the articles published previously. We should remember the tendency of these lesions to be located along the posterior aspect of the breast, which makes it challenging for MRI-guided biopsy. As discussed by Perlet et al. [40], the medial and posterior areas are considered “dead space”, and such a location can be a potential contraindication to MRI-guided biopsy. MRI surveillance for BRCA mutation carriers is especially important for early detection of posteriorly located lesions, as these are difficult to detect on both self-breast exam and clinical breast exam.

Our study has several limitations. First, this study was retrospective. Second, this was a single institutional study. Third, the image database we used was not acquired by one designated image protocol, because it included radiographic data patients from other facilities. Therefore, we need a larger population-based study in the future to improve and develop our study and the surveillance screening examination systems in Japan.

In this study, MRI-detected lesions were significantly smaller at the time of diagnosis than lesions detected by mammography and US. We have reported the first two MRI-detected lesions in high-risk females in Japan which underwent MRI-guided biopsy. There were no cases of breast cancer that were MRI negative and mammography positive. The results of this study suggest that in the near future, MRI screening and MRI-guided biopsy are likely to play important roles in the HBOC surveillance system established in Japan for high-risk women who have not yet developed breast cancer, especially since MRI-guided biopsy is covered by Japan’s national health insurance as of this year.
